# The Peptide Vaccine Combined with Prior Immunization of a Conventional Diphtheria-Tetanus Toxoid Vaccine Induced Amyloid *β* Binding Antibodies on Cynomolgus Monkeys and Guinea Pigs

**DOI:** 10.1155/2015/786501

**Published:** 2015-10-11

**Authors:** Akira Yano, Kaori Ito, Yoshikatsu Miwa, Yoshito Kanazawa, Akiko Chiba, Yutaka Iigo, Yoshinori Kashimoto, Akira Kanda, Shinji Murata, Mitsuhiro Makino

**Affiliations:** ^1^Iwate Biotechnology Research Center, 22-174-4 Narita, Kitakami, Iwate 024-0003, Japan; ^2^Venture Science Laboratories, R&D Division, Daiichi-Sankyo Co., Ltd., 1-2-58 Hiromachi, Shinagawa-ku, Tokyo 140-8710, Japan; ^3^Quality Assurance Division, Hayashibara Co., Ltd., 675-1 Fujisaki, Naka-ku, Okayama 702-8006, Japan; ^4^R&D Planning Department, R&D Division, Daiichi-Sankyo Co., Ltd., 1-2-58 Hiromachi, Shinagawa-ku, Tokyo 140-8710, Japan; ^5^Development Oversight Function, R&D Division, Daiichi-Sankyo Co., Ltd., 1-2-58 Hiromachi, Shinagawa-ku, Tokyo 140-8710, Japan; ^6^Biological Research Department, Drug Discovery and Biomedical Technology Unit, Daiichi-Sankyo R&D Novare Co., Ltd., 1-16-13 Kitakasai, Edogawa-ku, Tokyo 134-8630, Japan; ^7^Biological Research Laboratories, R&D Division, Daiichi-Sankyo Co., Ltd., 1-2-58 Hiromachi, Shinagawa-ku, Tokyo 140-8710, Japan

## Abstract

The reduction of brain amyloid beta (A*β*) peptides by anti-A*β* antibodies is one of the possible therapies for Alzheimer's disease. We previously reported that the A*β* peptide vaccine including the T-cell epitope of diphtheria-tetanus combined toxoid (DT) induced anti-A*β* antibodies, and the prior immunization with conventional DT vaccine enhanced the immunogenicity of the peptide. Cynomolgus monkeys were given the peptide vaccine subcutaneously in combination with the prior DT vaccination. Vaccination with a similar regimen was also performed on guinea pigs. The peptide vaccine induced anti-A*β* antibodies in cynomolgus monkeys and guinea pigs without chemical adjuvants, and excessive immune responses were not observed. Those antibodies could preferentially recognize A*β*_40_, and A*β*_42_ compared to A*β* fibrils. The levels of serum anti-A*β* antibodies and plasma A*β* peptides increased in both animals and decreased the brain A*β*_40_ level of guinea pigs. The peptide vaccine could induce a similar binding profile of anti-A*β* antibodies in cynomolgus monkeys and guinea pigs. The peptide vaccination could be expected to reduce the brain A*β* peptides and their toxic effects via clearance of A*β* peptides by generated antibodies.

## 1. Introduction

Alzheimer's disease (AD) is a neurodegenerative disease pathologically characterized by the deposition of the amyloid beta (A*β*) fragments derived from amyloid precursor protein (APP) in senile plaques and the accumulation of neurofibrillary tangles composed of tau protein [1, 2]. Increasing evidence suggests that accumulation of A*β* plays a central role in the onset and progression of AD, and therapeutic interventions have been directed toward the reduction of A*β* production using inhibitors of the *β*- and *γ*-secretase enzymes or enhancement of A*β* clearance by immunotherapy [3–5].

Regarding A*β* immunotherapy, both active immunization against A*β* and passive immunization with monoclonal A*β* antibodies were reported to attenuate amyloid plaque formation in the brains of APP transgenic mice [6–8]. These treatments also diminished the amyloid-associated pathology [9–11] and improved learning deficits [12, 13]. In the clinical trials of the AN1792 vaccine, the aggregated A*β*_1–42_ peptide with a QS-21 adjuvant, the long-term follow-up study analysis indicated that A*β*_1–42_ immunization resulted in clearance of amyloid plaques in patients with AD; however, this clearance did not lead to the prevention of progressive neurodegeneration [14]. In recent clinical trials, passive immunization with the anti-A*β* antibody, bapineuzumab and solanezumab, and intravenous immunoglobulin treatment failed to show a significant clinical benefit in patients with mild to moderate AD [15]. Although the clinical results were disappointing, there is a consensus in the field that A*β* immunotherapy by earlier intervention, targeting patients with early AD or mild cognitive impairment or presymptomatic subjects, could be an effective therapeutic and prophylactic treatment. Anti-amyloid combination therapies were also expected as practical approach for AD by the results that inhibition of *γ*-secretase or *β*-secretase with anti-A*β* antibodies was more effective than either alone in animal models [16, 17].

Based on the clinical results of the study of AN1792, which was halted due to the development of meningoencephalitis potentially related to a proinflammatory T-cell-mediated immune response [18–20], next-generation vaccine strategies for AD treatment will remain promising if the vaccine induces autoantibodies (anti-A*β* antibodies) without excessive inflammatory responses.

We have previously reported an A*β* peptide vaccine constructed of two parts, a T-cell epitope peptide on the N-terminal side and a B-cell epitope peptide connected by a dilysine linker (KK) to the C-terminal side of the peptide [21]. In order to enhance the immunogenicity of the peptide, a cell-attachment motif (RGD) was added to the N-terminal side of the peptide [21], and a multiagretope-type T-cell epitope was used for induction of antibodies to a wide range of MHC-II type individuals [22]. Although the A*β*_1–42_ peptide, the antigen of AN1792, is estimated to contain many T-cell epitopes including cytotoxic epitopes, the N-terminal region of A*β* was thought to be an effective and safer target [23–25]. Our vaccine contained only the A*β*_1–13_ as a target B-cell epitope peptide, which is estimated to contain few cytotoxic T-cell epitopes by* in silico* analysis [22]. Because the A*β*_1–13_ was as weak as the B-cell epitope, the utilization of the additional T-cell epitope peptide, recognizable by preexisting memory T-cells in the host, was necessary for induction of the antibody to A*β*_1–13_ [26]. We used the multiagretope-type T-cell epitope peptide from diphtheria toxin (DiTox_382–401_). The diphtheria toxin was used as a conventional vaccine antigen, such as diphtheria and tetanus (DT) vaccines designed to induce the toxin-neutralizing antibodies by Th2 type humoral immunities, and the major memory T-cells responding to DT epitopes were estimated to induce Th2 type immune responses. Our peptide vaccine, the RGD-DiTox_382–401_-KK-A*β*_1–13_ peptide, could induce the anti-A*β* antibodies to C57BL/6 by boosting the T-cell reaction preimmunized by DT vaccination without chemical adjuvants [26]. This result provided motivation to investigate whether our peptide vaccine will also be effective to other species.

In this study, we investigated the immunogenicity of the peptide with vaccination to cynomolgus monkeys and guinea pigs and studied the effects of antibodies by monitoring the A*β* peptides.

## 2. Methods

### 2.1. Peptides

A RGD-DiTox_382–401_-KK-A*β*_1–13_ peptide (RGD-AYNFVESIINLFQVVHNSYN-KK-DAEFRHDSGYEVH, the numbers following DiTox indicated the position of the amino acids on the precursor protein of diphtheria toxin including the 32-amino acids signal peptide), synthesized and verified by MALDI-TOF/MS as over the 95% purity, was obtained from Operon Biotechnologies K.K. (Tokyo, Japan). The single-letter universally accepted notation for amino acids is used throughout this text. Human A*β* peptide fragments used in this study were purchased from AnaSpec, Inc. (CA, USA).

### 2.2. Animals

The vaccination studies on male cynomolgus monkeys (3 to 4 years of age at the start of the study) were performed at Mitsubishi Chemical Medience Corporation (Shibaura, Tokyo, Japan). Male guinea pigs (Slc:Hartley) were purchased from Japan SLC, Inc. (Hamamatsu, Japan), and immunization began at 5 weeks of age. All experimental procedures were performed in accordance with the in-house guideline of the Institutional Animal Care and Use Committee of Daiichi Sankyo Co., Ltd.

### 2.3. Immunization

Cynomolgus monkeys were primed with 0.5 mL of absorbed diphtheria-tetanus combined toxoid (DT vaccine: The Kitasato Institute, Tokyo, Japan) three weeks before peptide immunization. The A*β* peptide vaccine was subcutaneously administrated with 0.5 or 2.5 mg/0.5 mL/head eight times every two weeks.

Guinea pigs were primed subcutaneously with 50 *μ*L/head of DT vaccine before the peptide immunization. Three weeks after the DT vaccination, guinea pigs were immunized subcutaneously with 200 *μ*g/200 *μ*L/head of RGD-DiTox_382–401_-KK-A*β*_1–13_ peptide solution or 200 *μ*L of vehicle (distilled water) for the control group. Four identical booster doses were given at 3-week intervals.

### 2.4. Sample Collection

Peripheral blood was collected every 2 weeks from the cynomolgus monkeys; then plasma and serum were prepared and stored at −20°C for the following experiments.

Approximately 100 *μ*L of blood was collected from the guinea pigs by tail bleeding one week after each peptide administration. Plasma samples were prepared by centrifugation and stored individually with a complete protease inhibitor cocktail (Roche Diagnostics K.K., Tokyo, Japan). One week after the last booster dose, cerebrospinal fluid (CSF) was obtained; then the animals were bled and plasma samples were prepared. CSF and plasma samples were prepared by centrifugation and then stored at −20°C. The brains were removed, frozen on dry ice, and stored at −80°C for an ELISA assay.

### 2.5. ELISA for Anti-A*β* Antibodies

Plates were coated with A*β*_1–42_ dissolved in distilled water and then washed with wash buffer (0.05% Tween 20 in phosphate buffered saline; PBS). Next, the plates were blocked with 1% Block Ace (Bio-Rad Laboratories, Inc., Hercules, CA, USA) in PBS at room temperature and then washed with wash buffer. Plasma samples were diluted 100- to 1000-fold. The autologous 2H8 mouse monoclonal anti-A*β* antibody (Thermo Fisher Scientific K.K., Yokohama, Japan) was used to generate a calibration curve for antibody titers. Each sample was applied to a well and incubated at 4°C overnight. After washing the plate, the wells were incubated with horseradish peroxidase- (HRP-) conjugated anti-mouse IgG and anti-guinea pig antibody (Sigma-Aldrich Japan, Inc., Tokyo, Japan) at 4°C for 2 h. Next, they were incubated with 2,2′-azino-di-[3-ethyl-benzothiazoline-6 sulfonic acid] diammonium salt (ABTS) substrate (Bio-Rad Laboratories, Inc.) at room temperature in the dark. After sufficient color development had occurred, 2 M phosphate buffer was added to stop the reaction. The absorbance of each well at 405 nm was measured with a spectrophotometer and antibody titers were then calculated.

ELISAs for antibody epitope-mapping were performed using the following A*β* peptides: RGD-DiTox_382–401_-KK-A*β*_1–13_, A*β*_1–13_, A*β*_1–40_, A*β*_1–42_, and A*β*_1–42_ fibrils (fA*β*_1–42_) as an immobilized antigen, and ELISAs were performed in the same way as described above. The preparation of fA*β*_1–42_ is described as follows: Lyophilized A*β*_1–42_ in PBS was incubated at 37°C for three days and then the resulting A*β* solution was centrifuged at 4°C for 10 min at 10,000 ×g; then the precipitated fraction was suspended in distilled water and used in this study.

### 2.6. ELISA for Brain and Plasma

The brains of guinea pigs were thawed on ice and then homogenized in 5 volumes (v/w) of 42% formic acid solution including protease inhibitors using a homogenizer and a sonicator and were incubated overnight at 37°C. The homogenates were centrifuged at 37,000 rpm for 60 min at 4°C (OptimaTM L-100XP, rotor 50.4Ti, Beckman Coulter, Inc., Tokyo, Japan) and the supernatants were neutralized with 11 volumes (v/v) of 1 M Tris solution and then centrifuged at 10000 rpm for 10 min at 4°C (himac CT13R, Hitachi Koki Co., Ltd., Tokyo, Japan). The supernatants were collected as a brain A*β* fraction.

Levels of A*β*_40_ and A*β*_42_ in brain and plasma samples were measured using a Human/Rat *β* Amyloid (40) ELISA Kit Wako II (Wako Pure Chemical Industries, Ltd., Tokyo, Japan), Human/Rat *β* Amyloid (42) ELISA Kit Wako, High-Sensitive (Wako Pure Chemical Industries, Ltd.), and Human Amyloid *β* Oligomers (82E1-specific) Assay Kit (IBL Co., Ltd., Gunma, Japan) and were used according to the manufacturer's instructions.

### 2.7. Detection of Cytokines from Peripheral Blood of Cynomolgus Monkeys

Plasma of cynomolgus monkeys at days 22 to 36, 64 to 78, and 106 to 120 was pooled and used for the ELISA. IL-2, IL-4, IL-10, and TNF*α* were measured by a Monkey ELISA Kit (Invitrogen) according to the manufacturer's instruction.

### 2.8. A*β*_1–42_ Toxicity Assay

The soluble A*β*_1–42_ peptide was added to the 7th day after passage of rat adrenal medulla derived pheochromocytoma, PC12 (Dainippon Sumitomo Pharma Co., Ltd., Tokyo, Japan), cell line at 0 to 2 *μ*M, and the viability of the cells was evaluated by AlamarBlue (Pierce, Thermo Fisher Scientific, Inc., Waltham, MA, USA) according to the manufacturer's instructions. The anti-A*β*_1–42_ monoclonal antibody (0.33 *μ*M of 6E10, abcam, Cambridge, USA) was used as a control for the protection of PC12 cells from cytotoxic A*β*_1–42_ peptide. The antibody (0.33 *μ*M of mouse IgG, Sigma-Aldrich, St. Louis, MO, USA) was used as a negative control. The serum and antibodies were preincubated with A*β*_1–42_ peptides at 4°C for 2 h, then added to the cells, and cultured for 24 h.

### 2.9. Statistical Analysis

Data were expressed as mean ± standard error (SE). Data from the passive avoidance test and the brain, plasma, and CSF A*β* levels were analyzed by one-way analysis of variance (ANOVA). Data from the splenic T-cell proliferation assay were analyzed by the one-way layout and multiple comparison method of Dunnett. SAS System Release 8.2 (SAS Institute Inc.) was used to perform all analyses and *P* values of less than 0.05 were considered to be statistically significant.

## 3. Results

### 3.1. Immunization of A*β* Peptide in Cynomolgus Monkeys

**Figure 1:**
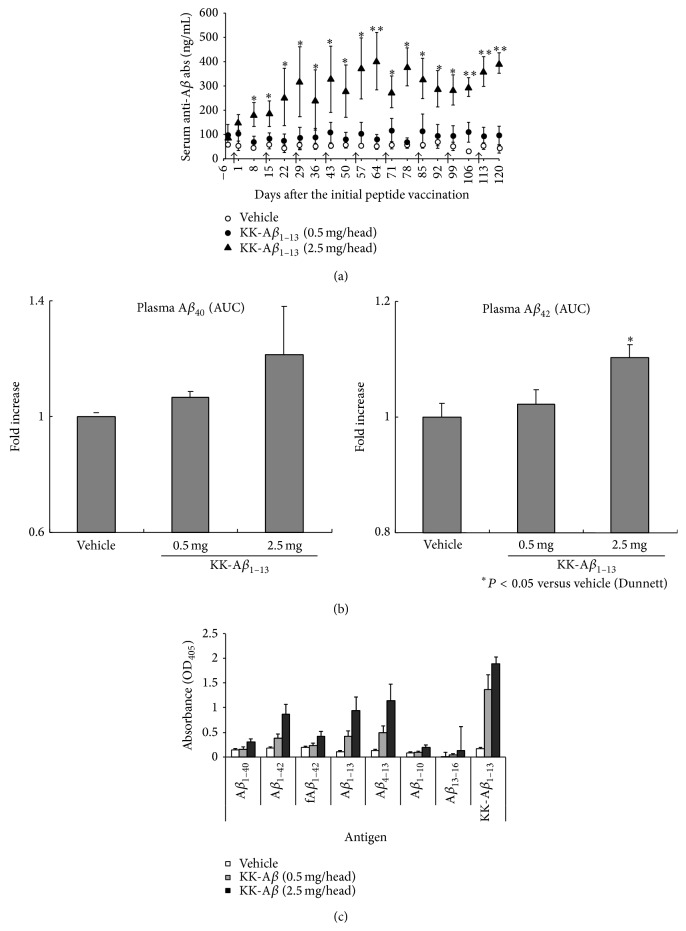
Induction of the anti-A*β* antibodies and A*β* peptides in the peripheral blood by immunization of the peptide vaccine to cynomolgus monkeys. The serum anti-A*β* antibody levels of the cynomolgus monkeys treated with vehicle or RGD-DiTox_20_-KK-A*β*_1–13_ (a). The plasma A*β*_40_ and A*β*_42_ peptide (b) level (nM) of cynomolgus monkeys. Diphtheria and tetanus toxoids (DT, 0.5 mL/head, s.c.) were administered to cynomolgus monkeys. Three weeks after the DT treatment, vehicle or the peptide (0.5 or 2.5 mg/head, s.c.) was administered at intervals of 2 weeks (arrows; total 9 times). Blood sampling was performed every week after the initial treatment. Results are represented as mean ± SE (*n* = 5). ^*∗*^ *P* < 0.05 and ^*∗∗*^ *P* < 0.01 as compared with the vehicle control group (the Dunnett test). Epitope-mapping of plasma anti-A*β* antibodies immunized with vehicle or RGD-DiTox_382–401_-KK-A*β*_1–13_ peptide (c). Epitope-mapping of antibodies was performed using each peptide-precoated ELISA with plasma collected at two weeks after the final treatment. Results are represented as mean ± SE (*n* = 5). KK-A*β*_1–13_: RGD-DiTox_20_-KK-A*β*_1–13_ peptide.

The time courses of plasma antibody concentration against the A*β* peptides by subcutaneous administration of the RGD-DiTox_382–401_-KK-A*β*_1–13_ peptide (0.5 and 2.5 mg/head) or vehicle (distilled water) in cynomolgus monkeys are shown in Figure 1(a). Compared to the vehicle group, the 2.5 mg peptide administrated group showed significantly higher antibody concentrations after the initial peptide administration (8 days after the first administration). To recognize the upper limit of the antibody concentration induced by the peptide, booster immunizations were continued over 100 days after the first immunization. The marked elevation following the third immunization was not observed and final serum antibody concentrations were reached about 8 times higher than that of the vehicle group. The plasma concentrations of A*β*_40_ were not significantly different between the groups, but the concentration of A*β*_42_ peptide in the 2.5 mg/head vaccination group was significantly higher than the vehicle group (Figure 1(b)). The result of epitope-mapping is indicated in Figure 1(c). The peptide induced antibodies recognized not only the A*β* vaccine peptide (KK-A*β*_1–13_) but also the full-length A*β*_1–40_ and A*β*_1–42_ peptide. The antibodies were less reactive to A*β* fibrils (fA*β*_1–42_) than A*β*_1–42_ peptide. The antibody reactivities against the A*β*_13–16_ and A*β*_1–10_ peptide were relatively weaker than that against the A*β*_1–13_ peptide, suggesting that the C-terminal side of the A*β*_1–13_ peptide was the main epitope of the antibody generated by this peptide vaccination.

The cytokine responses to the peptide immunization were investigated by ELISA. Plasma of the cynomolgus monkeys at days 22 to 36, 64 to 78, and 106 to 120 was pooled and used for ELISA. IL-2, IL-4, IL-10, and TNF*α* were measured, but all cytokines remained under the detection limits (Table 1; TNF*α* < 2 pg/mL, Il-2 < 2 pg/mL, Il-4 < 3 pg/mL, and Il-10 < 10 pg/mL).

### 3.2. Immunization of A*β* Peptide in Guinea Pigs

**Figure 2:**
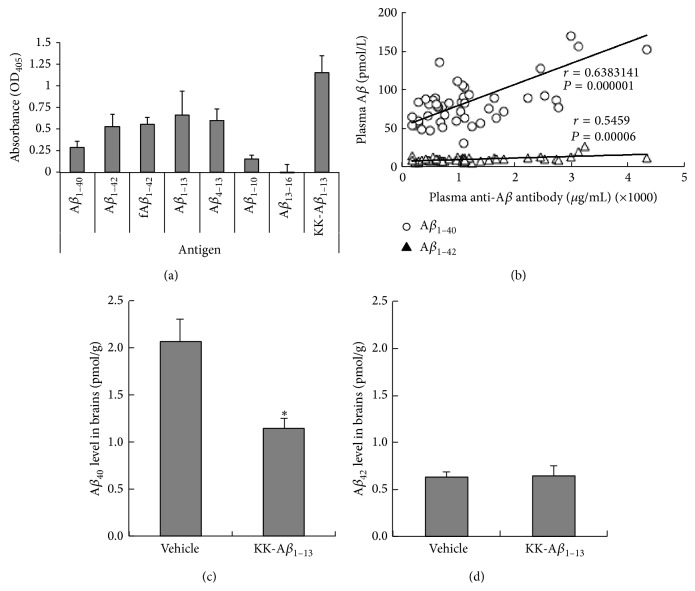
Induction of the anti-A*β* antibodies in the plasma and A*β* peptides in the brain by immunization of the peptide vaccine to guinea pigs. Epitope-mapping of plasma anti-A*β* antibodies immunized with RGD-DiTox_382–401_-KK-A*β*_1–13_ peptide (a). Epitope-mapping of antibodies was performed using each peptide-precoated ELISA with plasma collected at two weeks after the final treatment. Results are represented as mean ± SE. *n* = 5. The plasma anti-A*β* antibody levels of the guinea pigs treated with RGD-DiTox_20_-KK-A*β*_1–13_ peptides and the correlation of anti-A*β* antibody concentrations to A*β*_40_ and A*β*_42_ peptide doses in the plasma (b). The A*β* peptide levels in the brain of guinea pigs (c and d). Diphtheria and tetanus toxoids (DT, 200 *μ*L/head, s.c.) were administered to guinea pigs. Three weeks after the DT treatment, vehicle or the peptide (200 *μ*g/head) was administered at intervals of 3 weeks (arrows; total 6 times). Blood sampling was performed every week after the initial treatment. Results are represented as mean ± SE (*n* = 5). ^*∗*^ *P* < 0.05 and ^*∗∗*^ *P* < 0.01 as compared to the vehicle control group (the Dunnett test).

The A*β* peptide, RGD-DiTox_382–401_-KK-A*β*_1–13_ peptide (200 *μ*g/head), or vehicle (distilled water) was subcutaneously administrated in guinea pigs. Compared to the vehicle group, the peptide administrated group showed 6 to 8 times higher antibody titers throughout the experiment (data not shown). The epitope-mapping performed with the serum from the final bleed indicated that the peptide vaccination induced antibodies in guinea pigs in the same patterns of epitopes as in cynomolgus monkeys except that the antibodies were reactive to fA*β* as like as A*β*_1–42_ peptide (Figure 2(a)).

The concentration of the plasma A*β*_1–40_ peptide tended to increase due to the peptide immunization and showed a correlation with the level of anti-A*β* antibodies in the serum (Figure 2(b)). The concentration of the plasma A*β*_42_ peptide was ten times lower than A*β*_40_ peptide and their orders were unchanged by increasing anti-A*β* antibody concentration.

A*β*_40_ peptide levels in brains were significantly decreased by the peptide immunization group (Figure 2(c)), but the level of A*β*_42_ peptide did not change by immunization (Figure 2(d)).

### 3.3. A*β*_1–42_ Toxicity Assay with a Nerve Cell Culture

**Figure 3:**
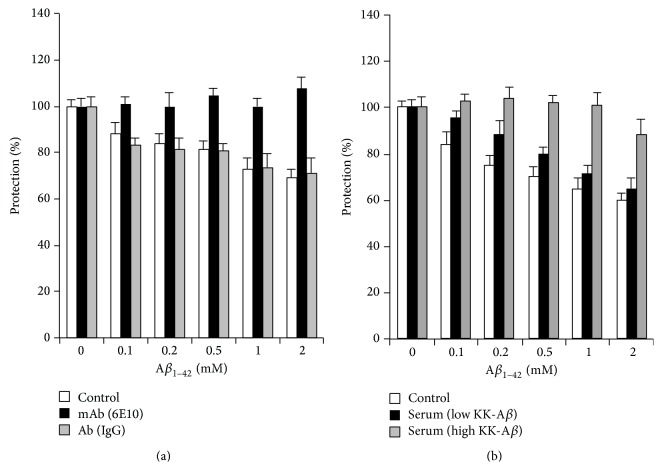
Effects of antiserum from cynomolgus monkeys vaccinated with RGD-DiTox_20_-KK-A*β*_1–13_ peptides or vehicle on A*β*-induced cytotoxicity of PC12 cells. The effects of the anti-A*β* monoclonal antibodies (positive control: black bar) and nonspecific immunoglobulin (gray bar) on the cytotoxicity of A*β* peptides (base control of cytotoxicity: white bar) are indicated (a). The effects of the serum of A*β*_1–42_ peptide-immunized monkey (0.5 mg/head low dose: black bar, and 2.5 mg/head high dose: gray bar) on the cytotoxic A*β*_42_ peptides (buffer control: white bar) are indicated.

To evaluate the protective effect of the peptide induced anti-A*β* antibodies, A*β*_1–42_ toxicity assay with a PC12 cell culture was performed. The anti-A*β* serum (25% final concentration) in cynomolgus monkeys collected one week after the final peptide immunization was investigated. Only the anti-A*β* antibody could protect PC12 cells from the toxic A*β* peptide (Figure 3(a)). The anti-A*β* serum of high-dose peptide-immunized monkeys protected PC12 cells from the damage of A*β*_1–42_ peptide (Figure 3(b)).

## 4. Discussion

The RGD-DiTox_382–401_-KK-A*β*_1–13_ peptide with preimmunized DT vaccine induced anti-A*β* antibodies in monkeys and guinea pigs without any chemical adjuvants. The vaccine peptide induced about 6 to 8 times higher anti-A*β* antibodies than vehicle-treated animals following third immunization. Those results clearly indicated the vaccine peptide was immunogenic to both kinds of animals. The multiagretope-type T-cell epitope peptide on the N-terminal side of our peptide worked as T-cell epitope of DT vaccine to the animals with many types of MHC [22], and the results of guinea pigs and cynomolgus monkeys showed the T-cell epitopes of each animal were included in the peptide.

The T-cell epitope sequence was derived from conventional DT vaccine for induction of antibodies to A*β* peptide. Davtyan et al. [27] reported the same concept of AD peptide vaccine, Lu AF20513, that used memory Th cells generated by tetanus toxoid vaccine for induction of A*β* antibodies. Lu AF20513 was immunized with strong adjuvants, CFA/IFA, Quil-A or Alhydrogel, and more than several hundred times higher titers of antibodies were induced. It supported the availability of our peptide vaccine, if the arrangement of the epitopes and the amino acid sequence of T-cell epitope were different from our peptide. Our peptide would also induce more strong immune responses immunized with strong chemical adjuvants (supplemental experiments; see Supplementary Material available online at http://dx.doi.org/10.1155/2015/786501). In this study, we tried to avoid the induction of the strong immune responses for reduction of the risk by unexpected immune responses, namely, cytotoxic responses reported in the clinical trial of AN1792 [14]. The excessive entry of antibodies in the brain also might be a risk of encephalitis. Following the 9 administrations of the peptide, significant levels of cytokines were not induced in cynomolgus monkeys (Table 1).

The result of the A*β*_1–42_ toxicity assay using PC12 cells presented the effectiveness of the anti-A*β* serum induced by the weak immune responses by our peptide vaccination. In addition, our peptide vaccination induced antibodies working for clearance of A*β* peptides from the brain of the guinea pigs (Figure 2) [7]. The epitope-mapping of antibodies of both animals indicated the antibodies commonly recognize the C-terminal side of the A*β*_1–13_, full-length A*β*_1–40_, and A*β*_1–42_ peptides (Figures 1(b) and 2(a)). A*β*_1–42_ fibril was also recognized by antiserum of guinea pigs but less reactive to antiserum of cynomolgus monkeys (Figures 2(a) and 1(c)). The antisera of our peptide would be more suitable for nerve cell protection from A*β*_1–42_ peptides than dissolution of the A*β*_1–42_ fibrils. This might be a major difference between AN1792 and our peptide. Lu AF20513 also used the A*β*_1–12_ peptide as B-cell epitope, and the epitope of the induced antibodies was thought to be common between Lu AF20513 and our peptide. The antibodies induced by our peptide significantly increased the plasma A*β*_1–40_ peptides and reduced the A*β*_1–40_ peptides in the brains (Figures 1 and 2). It would be considered that the anti-A*β* antibodies transport the A*β* peptides from the brain to the blood stream [28, 29]. In contrast, plasma and brain A*β*_1–42_ peptide was always lower than A*β*_1–40_ peptides and was not changed in the guinea pigs. It was expected that the basal level of A*β*_1–42_ peptides in the wild type guinea pigs was three times lower than A*β*_1–40_ peptide, and most of anti-A*β* antibodies would preferably react to A*β*_1–40_ peptide compared to A*β*_1–42_ peptide. Then it might be difficult to show the effects of the anti-A*β*_1–42_ antibodies in Figure 2(d).

We also investigated the effect of the peptide vaccine by immunization of Tg2576 mice expressing the Swedish mutation of APP (APPK670N, M671L) (supplements). Unfortunately, the antibodies were not induced without chemical adjuvants and Freund's incomplete adjuvant (FIA) was used three weeks after the DT vaccination. Tg2576 mice expressed excessive APPs in all tissues and strong adjuvants were necessary for induction of antibodies to A*β* peptides (Figure S1). The peptide vaccine could induce anti-A*β* antibodies to APP-Tg mice. The binding patterns of antibodies showed the same epitopes to guinea pigs and cynomolgus monkeys (Figure S1A). The results of the Tg2576 indicated that the vaccine induced antibodies increased A*β* peptides in plasma, which is consistent with the results in monkeys and guinea pigs, and CSF (Figures S1B, C, D, and E). The brain A*β* deposition and A*β* oligomer of APP-Tg mice reduced compared to vehicle-treated animals (Figures S1F, G, H, and I). Those results suggested that antibodies transported the A*β* peptides from the brain to the blood stream through the cerebrospinal fluid.

We investigated memory changes in a passive avoidance test after the last booster vaccination. Compared to non-Tg mice, the learning impairment had already been initiated in Tg2576 mice at the initial immunization (data not shown). Six months after the initial immunization, the learning impairment in mice treated with A*β* peptide was significantly less than that in mice treated with vehicle, suggesting that A*β* peptide vaccination slowed the progression of learning impairment. Biochemical analysis indicated a significant reduction of the levels of insoluble A*β*_1–40_, A*β*_1–42_, and A*β* oligomers in the brain of KK-A*β* peptide vaccinated mice. A significant reduction of A*β*_1–42_ immunostaining in the parietal cortex and hippocampus was also observed (data not shown) suggesting that the vaccination with the RGD-DiTox_382–401_-KK-A*β*_1–13_ peptide could have a potential to reduce and/or to inhibit the formation of senile plaques in the brains of Tg2576 mice. These results are similar to other findings of vaccinations [30, 31] and suggest that the vaccination with the RGD-DiTox_382–401_-KK-A*β*_1–13_ peptide might slow the progression of learning impairment mediated through the reduction of senile plaques, soluble A*β* oligomers, and/or inhibition of senile plaque formation in the brain. Those pharmaceutical effects, observed in Tg2576 mice, would be expected in wild type guinea pigs and cynomolgus monkeys.

## 5. Conclusions

RGD-DiTox_382–401_-KK-A*β*_1–13_ peptide vaccination in combination with a prior vaccination of the conventional diphtheria-tetanus combined toxoid vaccine induced anti-A*β*_40  and  42_ antibodies in cynomolgus monkeys and guinea pigs. It promoted A*β* clearance in the brains of guinea pigs. The peptide vaccination did not show any excessive immune responses in any tested animals. We propose that this peptide would be a possible therapeutic or prophylactic candidate for AD.

## Supplementary Material

(1) Aβ peptide vaccination in Tg2576 mice.
(2) Step-down passive avoidance test of Tg2576 mice.

## Figures and Tables

**Table 1:** Cytokines in the peripheral blood of cynomolgus monkeys.

	Th1 type		Th2 type	
	TNF*α*	IL-2	IL-4	IL-10
Control	n.d.	n.d.	n.d.	n.d.
0.5 mg/head-KK-A*β* peptide	n.d.	n.d.	n.d.	n.d.
2.5 mg/head-KK-A*β* peptide	n.d.	n.d.	n.d.	n.d.

Detection limits: TNF 2 pg/mL, Il-2 2 pg/mL, Il-4 3 pg/mL, and Il-10 10 pg/mL.

## Abbreviations

A*β*: Amyloid beta
DT: Diphtheria-tetanus combined toxoid
APP: Amyloid precursor protein
AD: Alzheimer's disease
CSF: Cerebrospinal fluid
PBS: Phosphate buffered saline
HRP: Horseradish peroxidase
BBB: Blood-brain barrier.
