# Supplementary material for: The Peptide Vaccine Combined with Prior Immunization of a Conventional Diphtheria-Tetanus Toxoid Vaccine Induced Amyloid β Binding Antibodies on Cynomolgus Monkeys and Guinea Pigs
Source: J Immunol Res. 2015 Oct 11;2015:786501. doi: 10.1155/2015/786501 (PMC4619934; doi:10.1155/2015/786501)
Supplement: Supplementary file 1 — (1) Aβ peptide vaccination in Tg2576 mice. (2) Step-down passive avoidance test of Tg2576 mice. [file 786501.f1.zip › description.docx]

Summary of the supplemental data

1, Aβ peptide vaccination in Tg2576 mice.

Aβ peptide immunization contribute to Aβ clearance in AD model Tg2576 mice. The immunization increased anti-Aβ antibodies and increased serum and CSF Aβ peptides. On the other hand, Aβ peptides in the brain were decreased.

2, Step-down passive avoidance test of Tg2576 mice.

Aβ peptide immunization contributed to memory maintenance of Tg2576 mice. Tg2576 mice decreased the score of step-down avoidance test than non-transgenic mice. Aβ peptide immunization improved the score of Tg2576 mice.
