# Supplementary material for: The Peptide Vaccine Combined with Prior Immunization of a Conventional Diphtheria-Tetanus Toxoid Vaccine Induced Amyloid β Binding Antibodies on Cynomolgus Monkeys and Guinea Pigs
Source: J Immunol Res. 2015 Oct 11;2015:786501. doi: 10.1155/2015/786501 (PMC4619934; doi:10.1155/2015/786501)
Supplement: Supplementary file 1 — (1) Aβ peptide vaccination in Tg2576 mice. (2) Step-down passive avoidance test of Tg2576 mice. [file 786501.f1.zip › NewSupplimentalFile150802.docx]

**Supplemental Data**

**Aβ peptide vaccination in Tg2576 mice.**

We examined whether the antibodies induced by Aβ peptide immunization contribute to Aβ clearance in Tg2576 mice. Female nontransgenic (non-Tg) and Tg2576 mice expressing the Swedish mutation of APP (APPK670N, M671L) were obtained from Clea Japan Inc. (Tokyo, Japan), and immunization began at 10-months old. Tg2576 mice were primed subcutaneously with 50 μL of DT vaccine before peptide immunization. Two weeks after the DT vaccination, 50 μg/ 50 μL/ head of RGD-DiTox_382-401_-KK-Aβ_1-13_ peptide solution or vehicle emulsified in an equal volume of incomplete Freund's adjuvant was administered intraperitoneally to the mice. Seven identical booster doses were given at 2-week intervals.

Our preliminary study indicated that Tg2576 mice generated Aβ peptide in all tissues and the immune responses against Aβ would decrease with tolerance. Thus, we administered the peptide with the Freund's incomplete adjuvant (FIA) to induce a strong immune response in this study, and the anti-Aβ peptide serum titers were about 8 times higher than the serum titer of vehicle immunized Tg-mice (data not shown).

The epitope-mapping analysed with the serum from the final bleed indicated that the antibodies in Tg2576 mice had almost the same patterns of epitopes as cynomologus monkeys and guinea pigs, except the reactivity to Aβ_1-42_ fibrils (Figure S1A).

The levels of Aβ_40_ and Aβ_42_ peptides in the plasma significantly increased in KK-Aβ_1-13_ peptide-administrated mice (Figure S1B and C). We also measured the level of the peptides in the CSF (Figure S1D and E). The concentration of peptides in the CSF of the peptide-immunized mice increased 3 to 4 times more than vehicle-immunized mice.

Then we examined the effects of the vaccination to the Aβ peptides in the brains of Tg2576 mice. The brains of Tg2576 mice were thawed on ice and homogenized in 5 volumes (v/w) of 1% CHAPS solution including protease inhibitors using a homogenizer and a sonicator. The homogenates were centrifuged at 37,000 rpm for 60 min at 4°C and the supernatants were collected as a soluble Aβ fraction. The remaining pellets were solubilized in 90% formic acid solution by sonication and were then incubated at 37°C overnight. The solutions were diluted in 9 volumes (v/v) of 90% formic acid and neutralized with 11 volumes (v/v) of 1 M Tris solution and then centrifuged at 10000 rpm for 10 min at 4°C. The supernatants were collected as an insoluble Aβ fraction.

The levels of insoluble Aβ_40_ and Aβ_42_ in the brains showed statistically significant decrease in Aβ peptide administrated mice (Figure S1F and G). The effects of anti-serum of the peptide immunized mouse on the brain Aβ oligomers also decreased (Figure S1H). The representative brain section stained with an anti-Aβ_42_ antibody and the stained plaque area of the peptide-immunized mice significantly decreased compared to vehicle control mice (Figure S3I).

**Figure S1-1**

**Figure S1-2**

**Figure S1** Aβ peptide vaccination in Tg2576 mice. Epitope mapping of plasma anti-Aβ antibodies induced by RGD-DiTox_401-382_-KK-Aβ_1-13_ peptide immunization (A). Epitope-mapping of antibodies was performed using each peptide-precoated ELISA with plasma collected at two weeks after the final treatment. Results are represented as mean ± SE. n = 5. Plasma and CSF Aβ_40_ (B, D) and Aβ_42_ (C, E) levels, insoluble Aβ_40_ (F), Aβ_42_ (G) and soluble Aβ oligomer (H) levels in the brain of Tg2576 mice were indicated. Results are represented as mean ± SE (n=22-24). **P < 0.01 and ***P < 0.001 as compared with the control (vehicle) group (Student’s t-test). KK-Aβ1-13 : RGD-DiTox_401-382_-KK-Aβ_1-13_ peptide. Results are represented as mean ± SE (n=22-25). *Arrows* beneath the graph indicate the time of each inoculation. The ratio of the area of the Aβ_42_ peptide in the representative brain sections of Tg2576 mice treated with vehicle or RGD-DiTox_401-382_-KK-Aβ_1-13_ peptide (C). KK-Aβ1-13 : RGD-DiTox_401-382_-KK-Aβ_1-13_ peptide.

***Step-down Passive avoidance test***

This behavioral test was performed one week after the last booster dose. The apparatus consisted of an acrylic box W250 x D210 x H240 mm with a stainless-steel grid floor. A platform W65 mm x D55 mm x H45 mm was placed in the center of the box. Electric shocks (0.5 mA) were delivered to the grid floor for 2 s with an isolated pulse stimulator. At the training trial, the mice were placed on the platform, and electric shocks were delivered when the mice descended to the grid floor. After 24-h training, the test trial was performed. The mice were placed on the platform and the time taken to descend to the grid floor was measured as step-down latency. Step-down latency was recorded for 5 min.

**Figure S2** The step-down passive avoidance test of the vaccinated Tg2576 mice. Cognitive performance of Tg2576 mice treated with vehicle or the RGD-DiTox_401-382_-KK-Aβ_1-13_ peptide in a step-down-type passive avoidance test. The test was performed at one week after the final treatment. Results are represented as mean ± SE (n=22-24). KK-Aβ_1-13_ : RGD-DiTox_401-382_-KK-Aβ_1-13_ peptide. **P < 0.01 as compared with the control (vehicle) group (Student’s t-test).
